# Development Boards and Microcontroller Platforms in Sports and Physical Activity: A Systematic Review

**DOI:** 10.3390/s26165027

**Published:** 2026-08-07

**Authors:** Boryi A. Becerra-Patiño, Miguel Andrés Peñuela-Arguello, Cristián David Zapata-Piratova, Aura D. Montenegro-Bonilla, Rodrigo Yáñez-Sepúlveda, José Francisco López-Gil, José Pino-Ortega

**Affiliations:** 1 Faculty of Physical Education, National Pedagogical University, Bogotá 480100, Colombia or boryialexander.becerrap@um.es (B.A.B.-P.); mapenuelaa@upn.edu.co (M.A.P.-A.); cdzapatap@upn.edu.co (C.D.Z.-P.); admontenegrob@upn.edu.co (A.D.M.-B.); 2 Faculty of Sport Science, University of Murcia, 30100 Murcia, Spain; josepinoortega@um.es; 3 Laboratory Faculty Education and Social Sciences, Universidad Andrés Bello, Viña del Mar 2520000, Chile; rodrigo.yanez.s@unab.cl; 4 School of Medicine, Universidad Espíritu Santo, Samborondón 092301, Ecuador; 5 Vicerrectoría de Investigación y Postgrado, Universidad de Los Lagos, Osorno 5290000, Chile

**Keywords:** experimental prototypes, real-world training, biomechanical variables, algorithms, athletic performance enhancement

## Abstract

**Background:** The development of data processing platforms makes it possible to monitor human behavior outside the laboratory, which facilitates decision-making regarding physical activity, health, and training. **Objective**: Analyze the available scientific evidence on data logging in the field of sports and physical activity using development boards and microcontroller platforms. **Materials and Methods:** A systematic review was conducted following the Preferred Reporting Items for Systematic Reviews and Meta-Analyses (PRISMA) guidelines. The selection and inclusion of studies in this review were based on the inclusion and exclusion criteria derived from the participants, interventions, outcomes (PIO) strategy. Methodological quality was assessed using the Methodological Index for Non-Randomized Studies (MINORS). **Results**: After all the information screening was completed, 23 documents met the eligibility criteria. The studies addressed a wide range of sports practices, including swimming, athletics, skiing, cycling, badminton, tennis, strength training, weightlifting, rehabilitation, outdoor route monitoring, and female contact-sport scenarios such as rugby, resulting in the implementation of microcontrollers in heterogeneous settings. **Conclusions**: For sports and health professionals, microcontroller-based systems can represent an effective way to implement objective and individualized monitoring, especially in high-performance contexts. For research, it is recommended that future studies focus on homogeneous comparisons with reference standards, larger and more diverse samples (including women), and longitudinal evaluations in real-world training and competition scenarios, so that these solutions evolve from functional prototypes into robust and transferable tools.

## 1. Introduction

Research on physical activity and sport has evolved from traditional physiological, biomechanical, psychological, and social approaches toward a paradigm that incorporates wearable technologies and electronic systems for the continuous acquisition of data in real-world environments. This transition was driven by the miniaturization of sensors, the proliferation of wireless communications, and the development of data processing platforms that allow monitoring human behavior outside the laboratory, facilitating decision-making in training, injury prevention, and sports health [1].

In this context, microcontroller platforms and development boards (e.g., Arduino, ESP32, Raspberry Pi, STM32) are characterized as accessible and versatile tools that integrate inertial, physiological, and environmental sensors. These platforms enable the reliable design of modular and cost-effective prototypes that can be adapted to the specific needs of sports-related research, facilitating experimentation and technology transfer to applied settings [2]. However, the heterogeneity of the solutions and the variability in design criteria and their respective calibration require rigorous evaluations of the quality of the data they produce [3].

The metrological validation and reliability of the sensors used in various sports applications are considered a priori; in such cases, the reliability and validity of the instruments are satisfactory: to give an example, regarding inertial measurement units (IMUs), the specialized literature shows that, for mean spatiotemporal parameters (e.g., pass times or stride length), validity and reliability can range from good to excellent standards [4]; on the other hand, indicators of variability or symmetry require stricter protocols and greater caution in their interpretation [5]. These findings highlight the need to evaluate each instrument’s configuration (hardware + firmware + algorithms) [6] against reference standards for quality and validity and to report on metrological indicators such as bias, root mean square error (RMSE), intraclass correlation coefficient (ICC) and Bland–Altman plots [7].

At the same time, ongoing advances in wearable biosensors are expanding the possibilities for obtaining relevant biochemical and physiological data to enhance athletic performance. In parallel, the development of recent technologies focused on sweat analysis enables non-invasive monitoring of biomarkers such as lactate, electrolytes, pH, and cortisol, providing data directly related to the athlete’s metabolic state, hydration, and response to physiological stress during exercise [8,9].

The integration of these sensors into wearable systems raises significant concerns regarding selectivity, stability, interference, and reproducibility, which must be addressed through informed approaches involving comparative validations and standardized quality controls grounded in academic research. Beyond individual signal capture, multimodal data fusion for example combining electrocardiography (ECG), peripheral oxygen saturation (SpO_2_) and motion can improve the accuracy of detection and classification of physiological and clinical events [10] and offers a promising framework for sports applications requiring complex inferences in real time [2]. The incorporation of deep learning models and multi-resolution fusion techniques facilitates the use of heterogeneous data sources; however, it introduces additional considerations regarding energy consumption, latency, and the need for high-quality, labeled data for training and validation [11,12].

Despite the potential, several methodological challenges limit the widespread adoption of solutions based on open microcontrollers and sensors in the professional sports field [13]. These include: the lack of agreed-upon standards for validating do-it-yourself (DIY) devices, the limited reporting of calibration and synchronization procedures, insufficient sample sizes in validation studies, and the lack of open and well-documented datasets that allow for reproducibility and comparison between studies [14]. These gaps make it difficult to assess the equivalence between low-cost prototypes and certified commercial devices, as well as to estimate the transferability of algorithms between different platforms [15].

Therefore, there is a clear need for a critical and systematic synthesis that: (a) identifies and classifies the most widely used open platforms in sports research; (b) evaluates the metrological quality and reliability of the signals obtained in different contexts of use; and (c) proposes homogeneous comparison protocols against commercial references and robust analysis methods. A structured review will allow for mapping evidence, identifying areas with promising results (e.g., IMU for mean gait parameters), and highlighting domains where uncertainty requires further research, such as the measurement of sweat biomarkers or the integration of real-time multimodal fusion [2,5,9].

Finally, directing future lines of work toward methodological standardization, data exchange, and interdisciplinary evaluation (engineering, sports science, and health sciences) will help microcontroller- and development board-based solutions transition from experimental prototypes to reliable tools in sports and clinical practice [16,17,18]. This collaborative approach will also facilitate the generation of rigorous evidence on efficacy, safety, and feasibility of implementation in real-world training and competition scenarios [19,20].

To date, no systematic review has been published that critically analyzes the use of microcontrollers and development boards. Therefore, the objective of this study was to synthesize scientific evidence related to the use of development boards and microcontroller platforms including Arduino, ESP32, Raspberry Pi, STM32, and their derivatives for data recording in sport and physical activity to establish reference patterns and contributions that can be used by different coaches and sports team professionals to verify the effect that training processes have on the adaptive responses of athletes and to provide researchers and institutions with a basis for promoting further studies on the application of these technological advances.

## 2. Materials and Methods

### 2.1. Design

A systematic review was conducted following the Preferred Reporting Items for Systematic Reviews and Meta-Analyses (PRISMA) guidelines [21] (Supplementary Material) and the guidelines for conducting systematic reviews in the field of sport science [22]. The PRISMA checklist was used to ensure transparency, accuracy, and quality in the publication of systematic reviews and meta-analyses. The search for documents considered the following guidelines: i) documents indexed in SCImago Journal Rank (SJR) and/or Journal Citation Reports (JCR) up to December 5, 2025; ii) the databases consulted were: Web of Science, Scopus, PubMed and SPORTDiscus; iii) other sources, such as and Google Scholar, ResearchGate and document citations, were consulted.

### 2.2. Inclusion and Exclusion Criteria

The two authors (M.A.P.-A. and C.D.Z.-P.) independently conducted the search and selection of information to avoid bias. Any disagreements (5% of the total documents) regarding the final inclusion/exclusion status were resolved through academic discussion, both during the selection and inclusion phases. During the discussion, the two independent authors simultaneously reviewed the articles according to the criteria established in the order shown in Table 1. This process was systematized in Excel. The academic discussions regarding study inclusion took into account the duplicate searches conducted by these authors on different days for document review, thereby reducing potential bias. All records were screened at the title and abstract level according to the predefined inclusion and exclusion criteria. Duplicates were initially identified and merged using reference management software.

Inclusion criteria were: (i) studies using microcontrollers, development boards, or embedded systems (Arduino, ESP32, Raspberry Pi, STM32, etc.) applied to sports, exercise, or physical activity monitoring; (ii) studies with IMUs or wearable sensors integrated into the platform for data acquisition/monitoring; (iii) studies with empirical results (validation, comparison with reference equipment, field tests) or clear descriptions of architecture/hardware/software. Exclusion criteria were: (i) theoretical articles without practical implementation or experimental data; (ii) exclusively optical systems (unless compared with IMUs/embedded systems); (iii) patents, conference abstracts without full text, commercial entries without an evaluable method; (iv) systematic reviews, meta-analyses, bibliometric analyses, narrative or literature reviews; and (v) articles written without peer review. For the selection and inclusion of studies in this review, a set of inclusion and exclusion criteria was established based on the population, interventions, and outcomes (PIO) strategy (Table 1).

Additionally, a standardized extraction matrix was designed using Microsoft Excel (version 18.0), collecting: (i) year of publication; (ii) country and discipline; (iii) type of microcontroller or board used; (iv) integrated sensors (inertial measurement unit [IMU], electromyography (EMG), heart rate (HR), pressure, etc.); (v) collected variables (physiological, biomechanical, environmental); (vi) sampling frequencies and calibration protocols; (vii) data transmission methods (Bluetooth, Wi-Fi, IoT, local storage); (viii) application context (laboratory, field, elite sport, health); (ix) sample size; (x) statistical methods used; (xi) comparison with commercial systems (if any); (xii) main results; (xiii) reported limitations.

### 2.3. Search Strategy and Data Extraction

Search strings were constructed by combining Medical Subject Headings (MeSH), Institute of Electrical and Electronics Engineers (IEEE) terms, and free-text keywords, using Boolean AND/OR operators: (“microcontroller” OR “Arduino” OR “ESP32” OR “Raspberry Pi” OR “STM32” OR “embedded system” OR “development board”) AND (“sport” OR “exercise” OR “physical activity” OR “biomechanics” OR “wearable sensor” OR “inertial measurement unit” OR “IMU”) AND (“data acquisition” OR “monitoring” OR “Internet of Things” OR “IoT”). Each database was adapted to its corresponding syntax. This search equation was used to identify studies in each database. Additionally, vocabulary control was performed for each keyword to improve the retrieval of documents in other languages. The searches were conducted to identify studies without other restrictions regarding publication date, language, or study design, provided they were experimental studies with results associated with the use of microcontrollers and development boards. In cases where it was not possible to obtain the full texts of the identified studies through institutional or open access subscriptions, the corresponding authors were contacted directly via the ResearchGate platform and/or institutional email, as suggested by other studies that have implemented this methodology [23].

### 2.4. Methodological Quality

The methodological quality of the included studies was assessed using the Methodological Index for Non-Randomized Studies (MINORS) [24]. The instrument consists of eight items for non-comparative studies, with a maximum score of 16 points, and four additional items for comparative studies, for a total of 12 items and a maximum score of 24 points. Consequently, items 1–8 were assessed in all 23 included studies, while items 9–12 were applied only to studies with a comparative design and were recorded as “not applicable” (NA) in non-comparative studies. The “Points” column corresponds to the sum of the scores obtained for the applicable items, excluding NA values. Each item scored 0 points when not reported, 1 point when reported but inadequately or incompletely, and 2 points when reported adequately (Table 2).

Quantitative analysis of the MINORS scale revealed varying methodological quality depending on the study design. In non-comparative studies (n = 18), the mean score was 9.84 ± 1.80 out of a maximum of 16 points, equivalent to 61.5% of the maximum possible score, with values ranging from 6 points [34] to 13 points [27,42]. In contrast, the comparative studies (n = 5) obtained a mean score of 12.80 ± 3.35 out of a maximum of 24 points, equivalent to 53.3% of the maximum possible score, with scores ranging from 8 points [26,39,43,44] to 16 points [31]. Individual analysis of the items revealed high consistency in certain methodological aspects. Items 1 (clearly defined objective) and 4 (variables or outcomes appropriate to the objective) achieved the maximum score of 2 points in all 23 studies (100%). Likewise, item 6 (follow-up period appropriate to the study objective) received the maximum score in 20 of the 23 studies (87.0%) and a score of 1 or 2 points in all 23 studies (100%). In contrast, recurring methodological limitations were identified in the items related to follow-up losses and sample size calculation. Both item 7 (follow-up loss <5%) and item 8 (prospective sample size calculation) scored 0 points in 22 of the 23 studies (95.7%). Taken together, these results suggest that, although the studies demonstrate strengths in defining objectives and selecting outcomes, there is limited reporting on aspects related to participant follow-up and to prospective sample size calculation.

### 2.5. Data Synthesis

Since this review focused on synthesizing the scientific evidence related to the use of development boards and microcontroller platforms including Arduino, ESP32, Raspberry Pi, STM32, and their derivatives for recording data related to sports and physical activity, no statistical effect measures or additional analyses were applied. The parameters related to development boards and microcontroller platforms including Arduino, ESP32, Raspberry Pi, STM32, and their derivatives used for data logging were synthesized using a structured technical classification. To account for methodological heterogeneity and the absence of standardized outcome measures, methodological quality was assessed using MINORS, as described above. This approach aimed to ensure clarity and transparency in the document selection process, thereby avoiding potential biases related to measurement and data processing that were relevant to the methodological objectives of this review.

To mitigate the inherent technological and methodological heterogeneity of the studies, the comparisons were not based on absolute and/or definitive quantitative results but rather on a systematic functional and architectural categorization structured within the conceptual framework of the PIO strategy (Table 1). This paradigm provides a qualitative overview of how various platforms address common instrumentation challenges (sensor integration, data processing, and transmission) in physical activity, sports disciplines, etc. However, this technical diversity introduces limitations on the external validity and generalizability of the conclusions. On the one hand, the marked difference in efficient sampling rates which are sufficient for low-data-transfer-rate applications (e.g., Arduino boards for step counting) but critical for demanding biomechanical analyses that require real-time edge computing to prevent data loss (e.g., ESP32 or STM32 platforms) prevents a direct comparison of performance. Furthermore, the lack of standardized calibration protocols and the divergence between controlled laboratory trials and ecological field conditions restrict the universal transferability of the results. Consequently, the compiled findings are discussed with caution, interpreted as proof-of-concept solutions tailored to specific sports-related constraints rather than as absolute reference standards for instrumentation.

## 3. Results

### 3.1. Identification and Selection of Studies

**Figure 1:**
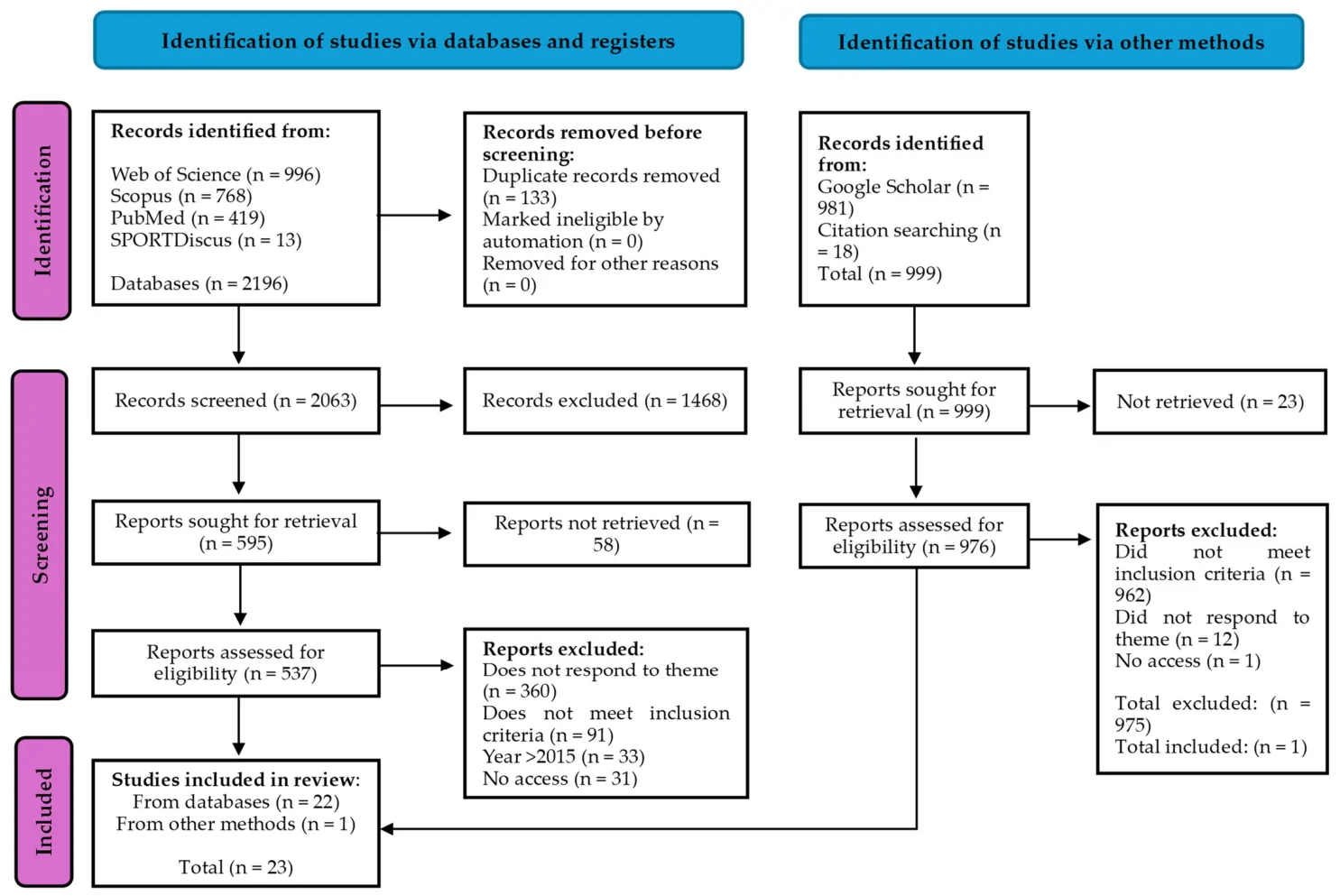
Flow diagram of the systematic review.

The database search identified 2196 records (Web of Science, n = 996; Scopus, n = 768; PubMed, n = 419; and SPORTDiscus, n = 13). After 133 duplicate records were removed, 2063 records were screened by title, abstract, and keywords, and 1468 were excluded. Of the 595 reports sought for retrieval, 58 could not be retrieved; therefore, 537 database reports were assessed for eligibility. A further 999 records were identified through other methods (Google Scholar, n = 981; citation searching, n = 18); 23 reports were not retrieved and 976 were assessed for eligibility. After full-text assessment, 515 database reports and 975 reports identified through other methods were excluded. Consequently, 23 studies met the eligibility criteria and were included in the review (22 from databases and 1 from other methods) (Figure 1).

### 3.2. Analysis of the Studies

**Figure 2:**
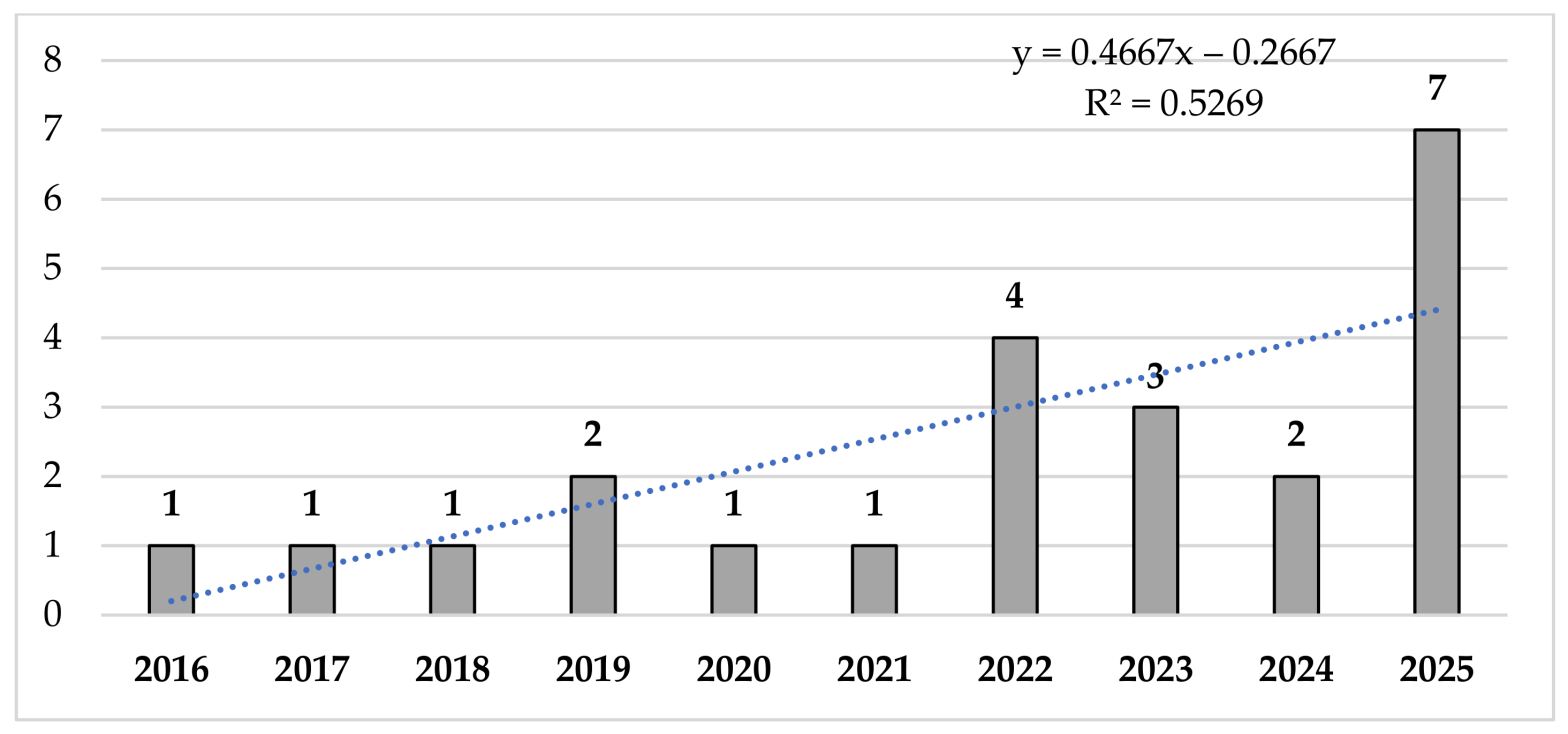
Number of studies published per year.

The graph shows the annual trend in the number of publications between 2016 and 2025. A fluctuating trend is observed during the early years: one publication in 2016, none in 2017, and one to two publications per year from 2018 through 2021. Starting in 2022, a significant increase is evident, reaching four publications in 2022, third in 2023, followed by a decrease to two in 2024 and a peak of seven publications in 2025. An ordinary least-squares trend line fitted to the annual counts for 2016–2025 (x = 1…10) has a positive slope (y = 0.4667x − 0.2667), indicating overall growth in scientific output over the analyzed period, although with moderate explanatory power (R^2^ = 0.5269) (Figure 2).

### 3.3. Analysis of the Participants

The 23 studies included in this systematic review comprised 143 explicitly reported human participants, one proof-of-concept prototype, and seven additional studies in which the exact number of athletes/respondents was not reported [27,28,30,33,34,40,45]. The total of 143 was calculated by summing only explicit person-level sample sizes in Table 3; non-person observations (e.g., pitches or kick samples), the proof-of-concept prototype, and studies reported as NR were excluded. For studies that reported both recruited and analyzed subsamples, the total sample recruited was counted once (e.g., 11 healthy adults in Smiley et al. [36] and 4 female participants in Fay et al. [47]). Table 3 specifies the characteristics of each included study (country, discipline, sample, and principal objective).

Similarly, Table 4 separates the processing architecture and the different sensor ecosystems used in the included studies to establish hardware reference patterns.

Table 5 details the prevalence of the implementation of wearable technologies, analyzing the system’s intelligence to transform raw signals into valid performance metrics through computational models. This approach allows for the analysis of technical gestures and the monitoring of physical variables in real time, determining that local processing is viable for feedback without depending on external servers.

The effectiveness and efficiency of microcontroller applications in the context of athletic performance enable coaches, scientists, and various stakeholders involved in sports across different disciplines to make informed decisions, thanks to their validated parameters. The findings of the included studies demonstrate how physical variables can be evaluated with minimal errors compared to laboratories that analyze movement based on its mechanical description. Table 6 analyzes Biomechanical/functional variables measured and main results and thus examines the operational applicability of microcontrollers in specific sports performance contexts. The various findings suggest that, despite the high precision, portability remains a determining factor for successful implementation in the different sports where the studies were conducted.

Finally, Table 7 analyzes a design pattern based on the portability and connectivity of devices using Bluetooth Low Energy (BLE) and Wi-Fi. These are established as the primary communication standards, facilitating real-time data transfer with very low latency. Furthermore, the robustness of the software demonstrates that the ease of use of these boards allows the implementation of distributed architectures, from basic Arduino signal transmission to Android-based systems for wireless monitoring and response-time feedback.

## 4. Discussion

The selected studies were conducted between 2016 and 2025. Regionally, there is significant research activity in Europe and Asia, with additional representation in Oceania through recent work from Australia. The studies addressed a wide range of sports practices, including swimming, athletics, skiing, cycling, badminton, tennis, strength training, weightlifting, rehabilitation, outdoor route monitoring, and female contact-sport scenarios such as rugby, resulting in the implementation of microcontrollers in heterogeneous settings. Thus, the results obtained were classified according to hardware architecture and the variables measured by microcontrollers performing various functions quantification, monitoring, and evaluation, thereby establishing reference standards for accuracy and agreement. Consequently, this review reveals that the use of open-source microcontrollers and development boards in the context of physical activity and sports is still in an early stage of consolidation, rather than in large-scale confirmatory studies; this stage is characterized by a clear emphasis on the development of various prototypes, proof-of-concept testing, and their preliminary validation.

One striking finding is that most of the included studies did not have objectives related to performance intervention; rather, they focused on the development, implementation, or validation of measurement systems. In this regard, many studies describe the design of portable platforms for acquiring kinematic or dynamic variables [29,34,35,46]; on the other hand, other studies take a more metrological approach by comparing their devices with reference instruments or commercially established systems [26,36,40]. This type of study is reported to make a significant contribution to measurement and monitoring, rather than to the direct optimization of sports training processes.

From a technological standpoint, there is a clear preference for and widespread use of open-source, low-cost platforms, particularly ESP32 and Arduino, over solutions based on Raspberry Pi and STM32, which are implemented less frequently. Although the results merely confirm this usage trend, the interpretation reveals that these platforms facilitate an accessible rapid prototyping ecosystem, utilized by both researchers and sports professionals, which aligns with findings from studies focused on the development of portable and wearable systems [35,42,47], as well as in compact IMU-based systems configured for the real-time characterization and classification of exercise technique from a biomechanical perspective [46]. This finding can be interpreted because the tools typically used in specialized laboratories primarily in applied contexts are relatively inexpensive and flexible solutions. Regarding the types of parameters analyzed, the included studies show a predominance of biomechanical measurements, specifically kinematic, accelerometric, force, localized impact, and execution speed variables [26,34,40,47].

Similarly, other systems also incorporated variables for monitoring cadence and cardiorespiratory parameters such as heart rate and oxygen saturation in telerehabilitation settings targeting cyclists [36]. On the other hand, there is a somewhat more limited presence of physiological or psychophysiological variables, as well as an integration of the tactical, cognitive, or contextual dimensions of athletic performance, which appear to be almost nonexistent. Therefore, it can be concluded that, to date, the use of microcontrollers in the field of sports has developed primarily as an extension of classical biomechanical analysis instrumentation and its considerations, rather than as a tool for the comprehensive evaluation of the athlete’s performance.

An important consideration is the level of validity and reliability demonstrated by the studies, which compare these systems with standard reference instruments on the market. Some studies show high correlation coefficients, high ICC values, acceptable errors, or robust calibration adjustments, indicating that at least under controlled conditions these analyzed devices can achieve levels of accuracy comparable to those of high-quality laboratory solutions [26,36,40,47]. Other studies, such as that by Navarro-Iribarne et al. [34] contribute from the perspective of technical development and functional demonstration, providing real-time visualization of kinematic traces rather than formal validation against another similar reference device.

This highlights a significant gap between technological development and its application in sports performance contexts; however, sports involving water-based elements, outdoor settings, or women’s contact sports have already been explored in more applied contexts [32,35,47]. Nevertheless, evidence based on longitudinal control studies, large samples, or competitive settings remains limited. Therefore, the potential of these technologies to enhance or transform daily monitoring of sports training continues to depend on a greater accumulation of studies with more robust designs and a higher degree of external validity.

From an applied research perspective, the results of this review indicate that open-source microcontrollers and development boards should not be viewed as direct substitutes for established commercial systems, but rather as highly valid complementary alternatives, useful in contexts with financial constraints, in applied research, or during the prototyping and methodological development phases. In this regard, studies such as those by Mohammed et al. [29], Duarte et al. [46], and Fay et al. [47]; highlight the potential of these possible solutions for designing and adapting specialized systems tailored to the specific needs of real-world sports scenarios needs that are difficult to meet with commercial products.

### 4.1. General Characteristics of the Studies

The total sample of documents for this review consisted of 23 included studies, all of which were published between 2016 and 2025, revealing a gradual increase in interest in microcontrollers and development boards used for data recording, storage, and presentation in the field of physical activity and sports. The studies were conducted primarily in Europe (Spain, Portugal, Romania, Slovenia, Turkey), Asia (China, Taiwan, Indonesia, Korea, Jordan), and Oceania (Australia), with a smaller number in North America. The contexts of application were varied and included swimming, soccer, multi-sport activity classification, athletics, badminton, running and health monitoring, skiing, discus and hammer throwing, cycling, home telerehabilitation, outdoor route monitoring, strength training and weightlifting, rehabilitation, tennis, martial arts, and women’s contact sport/rugby. This diversity reflects the versatility of the integrated systems for use in multiple sports contexts.

For its part, sample size showed considerable variability, ranging from case studies (n = 3), studies with samples of more than 40 participants and studies in which the exact number of athletes or respondents was not explicitly stated [27,28,30,33,34,40,45]. At the same time, studies with small samples (≤ 20 subjects) focused on the technological validation of the instrument. Likewise, the participant data from the studies also showed limited representativeness in terms of gender, with male or mixed/unspecified samples, and only a few isolated studies explicitly focused on female samples, such as in Fay et al. [47]. Finally, the main objectives focus on validating theoretically low-cost devices against commercial reference systems, in addition to developing prototypes for real-time monitoring and automatically classifying gestures or technical elements using precise algorithms.

### 4.2. Microcontrollers and Hardware Architecture

The most widely used microcontroller platforms in their different versions were ESP32 and Arduino, while Raspberry Pi-based solutions (including Raspberry Pi 4 and Pico W), STC89C52-based controllers, and hybrid configurations with STM32 were used less frequently. Among the studies included, the ESP32 was one of the most recurrent development boards in portable and wearable systems. Its use was mainly observed in applications oriented toward real-time biofeedback, gesture recognition, kinematic monitoring, and speed-based training control systems. In contrast, Arduino boards were used more frequently in simpler or laboratory configurations, while Raspberry Pi platforms appeared in computer vision, multisensor monitoring, and specialized wearable force-sensing architectures.

A wide variety of microcontrollers were implemented in different scientific studies; however, the most used microcontrollers were the ESP32 and Arduino. Studies that implemented the ESP32 used platforms that were dominant in the studies, emphasizing the presence of integrated Bluetooth and Wi-Fi signals, allowing real-time signal transmission (IoT) and the processing of integrated Machine Learning elements (Edge AI) [40,42]. In contrast, Duarte et al. [46] employed a Seeed Studio XIAO nRF52840 Sense board with integrated BLE and a 6-axis IMU to classify the correctness of weightlifting exercises directly from the weight being handled.

Thus, using ESP32 microcontrollers, load cells and rotary/linear encoders could be integrated, which is the standard for this type of variable. This demonstrates the validity of the criteria implemented by microcontroller-based platforms. For example, low-cost rotary encoders found reduced systematic errors in the measurement of mean propulsive velocity (MPV) in strength-focused training [40]. In parallel, Arduino-based IMU systems have enabled the real-time reconstruction of 3D wrist trajectories and movement speed in technical gestures such as the discus throw [34]. In addition, the processing capacity of the ESP32 has enabled IMU-based vibrotactile systems for real-time correction of swimming strokes, combining Dynamic Time Warping with local feedback during front-crawl execution in water [44]. These findings confirm that the use of microcontrollers provides a rigorous, low-cost, and high-precision alternative for real-time data tracking in sports, not only for monitoring physical variables but also for developing devices such as the balance board [41]; monitoring can be developed for physical therapists and rehabilitation specialists through objective monitoring and visual feedback for sports recovery, especially in environments that are considered purely clinical.

For their part, Arduino Nano and Arduino Uno microcontrollers were notable in initial studies for their ease of programming and low cost. In parallel, other low-cost controllers such as the STC89C52 were incorporated into distributed court-based systems for agility assessment, using photoelectric triggering and Wi-Fi communication to record reaction time in badminton-specific tasks [37]. This diversity in platforms allows for a certain degree of versatility in microcontroller-based designs. In another line of research within the field of home-based telerehabilitation, Smiley et al. [36] employed an ESP32-based architecture to monitor and control exercise components, demonstrating that low-cost wireless interfaces can achieve sufficient accuracy for tracking cadence.

It is noted that the Arduino Uno and Nano were used as the foundation for low-cost data acquisition in the initial studies, which focused primarily on interface simplicity. In addition, distributed embedded systems were also used that combined specific controllers, such as wireless communication modules and Android applications, which enabled real-time feedback without relying on a highly complex laboratory [37]. Similarly, in rehabilitation-focused contexts, ESP32-based systems were integrated with reed switches, Hall sensors, and tablet applications to display real-time cadence, heart rate, and oxygen saturation variables during cycling workouts; and remote speed control was enabled on motorized equipment [36]. Arduino-based wearable systems equipped with wrist- and ankle-mounted inertial sensors have also been used for multi-sport recognition through deep learning. Hsu et al. [27] implemented two six-axis sensing modules connected by RF transmission and reported very high recognition rates for ten sport actions after transforming acceleration and angular velocity signals into spectrograms processed with CNN.

With these microcontrollers, one of the most striking findings for sports professionals is the significant correspondence that can be achieved between low-cost systems and reference instruments. Smiley et al. [36], for example, reported small mean differences in cadence (RPM) between their cycling interfaces and a laser tachometer, especially in BLE and Wi-Fi modes, which supports the feasibility of remote monitoring in home telerehabilitation. Likewise, Pousibet-Garrido et al. [40] validated the use of a wireless encoder to monitor velocity-based training (VBT), while Navarro-Iribarne et al. [34] showed that an Arduino-based wearable IMU can generate technically useful visual feedback on wrist trajectory and speed during discus throwing. Raspberry Pi-based solutions were less frequent, but they supported computationally demanding tasks and specialized wearable architectures, ranging from multisensor physiological monitoring in cycling and on-node computer vision for detecting users on outdoor trails to high-frequency wearable force sensing for breast-impact monitoring in female athletes [35,39,47].

In the cycling-oriented implementation by Duran Bernardes and Ozbay [35], this microcontroller facilitated the monitoring and quantification of physical variables in real time, such as the recording of blood oxygen saturation (SpO_2_) through an integrated pulse oximeter and heart rate (HR). In turn, it monitored performance metrics by recording pedaling cadence and power in watts. This microcontroller was used to process complex power data that cannot be obtained with a simple pulse sensor. Thus, while the ESP32 is primarily used in lightweight, low-power portable devices, the Raspberry Pi is essential for complex monitoring stations that require greater processing power, various connection and communication protocols, or local computer vision workloads.

### 4.3. Integrated Feature Sensors and Estimated Variables

The most used sensors were IMUs, followed by load cells, rotary or optical encoders, GPS sensors, pressure sensors (FSRs), photoelectric sensors, cameras used for integrated machine vision, and physiological biosensors (heart rate, body temperature, and SpO_2_). IMUs, consisting of 6 or 9 axes, enabled the recording and storage of biomechanical variables such as acceleration, angular velocity, orientation, movement frequency, joint angles, and 3D kinematics, used in various sports such as swimming, discus throw, skiing, weightlifting, tennis, and martial arts [33,34,42,45,46]. These variables were used for kinematic analysis, as well as for the automatic and systematic classification of technical movements. In contrast, thin-film strain sensors (FSRs) enabled the analysis of localized impact forces at chest height and their spatial distribution in women’s contact sports [47], while camera-based systems facilitated the detection and counting of different trail users in outdoor sports [39].

Load cells and encoders were primarily used to estimate variables such as force, reaction time, power, and average propulsion speed in contexts related to strength training and the assessment, monitoring, and control of performance [31,40]. On the other hand, GPS sensors were used to determine trajectory and quantify external load, particularly in field settings. In parallel, a few studies were identified that incorporated physiological sensors and cadence monitoring modules, which allowed for real-time recording of heart rate, oxygen saturation, and pedaling cadence [36], primarily in applications focused on health, effort control, and rehabilitation in a synchronous manner.

### 4.4. Metrological Results: Validity, Precision, and Reliability

Metrological results are central to the documents analyzed and included. Several works compared microcontroller-based systems with commercially available instruments considered the gold standard, reporting metrics for concurrent validity, precision, and reliability. The measurement of biomechanical and operational variables showed high levels of agreement. In cycling telerehabilitation systems supported by ESP32 interfaces, mean differences between cadence values recorded by the device and those obtained with a laser tachometer remaining low, which supports the accuracy of BLE and Wi-Fi communication modes and an acceptable performance in motorized Bluetooth control [36]. Similarly, velocity-based training (VBT) systems showed very high correlations (r = 0.98) with commercially available reference devices, along with low mean absolute errors [40].

In contrast, Navarro-Iribarne et al. [34] contributed a functional validation centered on the real-time graphical representation of wrist trajectory and speed during discus throwing, without reporting a formal comparison against a commercial gold-standard system. Miguel et al. [39], in turn, reported a final mean average precision of 78.4% for YOLOv3-Tiny and a successful end-to-end transfer of detected records from the trail node to the central database, supporting the feasibility of the proposed route-monitoring architecture. It has been established that, in applications involving kinematic characteristics, measurements of joint angles, movement frequency, and movement patterns showed high correlations (r > 0.88) compared to video analysis systems or other reference methods. In the field of technical gesture classification, algorithms implemented on microcontrollers achieved an accuracy of between 94% and 98%, demonstrating a significant ability to distinguish various sports actions.

### 4.5. Guidelines or Recommendations for Selecting Microcontroller and Sensor Platforms Suitable for Data Logging in Physical Activity and Sports

Based on the findings of this review, we suggest considering the following guidelines when selecting microcontroller and sensor platforms for data logging:

- *Objective and key metrics to be measured.* It is essential to clearly define what is to be measured, for example, to assess movement (accelerometer/gyroscope), heart rate (ECG/PPG), muscle activity (EMG), position, speed, acceleration, and distance traveled by athletes in real time (GPS). Additionally, it is necessary to establish the priority of the measurement application, that is, whether it is for training, performance, rehabilitation, etc.
- *Sampling and accuracy requirements*. It is essential to determine the sampling frequency required for the measured variable; for example, 50–200 Hz for accelerometers and > kHz for EMG measurements. Furthermore, it is necessary to consider the sensor’s accuracy and/or noise and resolution, such as bits or dynamic range.
- *Synchronization and latency.* It is necessary to establish synchronization among multiple sensors (hardware timestamping, synchronization via PPS/GPS, or a protocol such as PTP). Likewise, define the latency tolerable by the system for real-time processing or feedback.
- *Power and battery life.* It is essential to analyze power consumption during active use and at rest, which allows for determining the actual application runtime. This also helps determine low-power modes and sleep options.
- *Connectivity and storage.* Here, it is necessary to understand the communication system: BLE (common in wearables), Wi-Fi, ANT+, LoRa, USB; local storage via microSD or flash memory if data is not transmitted in real time; and bandwidth for raw data (e.g., high-frequency IMU).
- *Processing and capacity.* Determine whether processing is done locally (filtering, event detection, ML) or if raw data is transmitted. This allows you to select an MCU with the appropriate CPU/FPGA/TPU. Additionally, ensure sufficient RAM/Flash memory for buffers and temporary storage.
- *Form factor and mounting.* Understand key characteristics such as size, weight, ergonomics, mounting method (strap, clip, clothing, etc.), and ruggedness and comfort for athletic use (sweat resistance, chafing).
- *Environmental ruggedness.* Protection mechanisms (water/sweat resistance), temperature range, vibration, and shock resistance, among others.
- *Software compatibility and community. Determine whether you need SDKs, libraries (MATLAB R2026a, Massachusetts, USA; Python 3.14.6, Oregon, USA; TensorFlow Lite v2.21.0, California, USA), driver support, and examples, as well as an active community and documentation to accelerate development.*
- *Regulation, security, and data privacy.* Compliance with regulations if biomedical data is involved, for example, for clinical use as well as encryption during transmission and storage, and consent policies.
- *Cost and scalability.* Determine the unit cost for prototypes and production, as well as the ease of mass production and long-term availability of components.
- *Validation and reliability.* Review the pilot tests against reference standards (motion cameras, clinical-grade ECG) to verify validity and establish a quality control plan and robustness tests for the data obtained.
- *Practical platform recommendations.* Determine the need for prototypes or low-cost solutions: Arduino (SAMD), Raspberry Pi Pico, ESP32 (BLE/Wi-Fi), and/or wearables with BLE and sensor support: Nordic nRF52 (low power consumption). Likewise, applications with high processing/ML demands: STM32H7, Raspberry Pi (for edge computing), and validated commercial solutions.
- *Selection of common sensors.* Select the sensor based on your needs; (i) IMU: Bosch (BMA/BSX), InvenSense (ICM/MPU) to verify range and noise; (ii) integrated accelerometers/gyroscopes/compasses (9–18 DOF) for full motion tracking; (iii) ECG: low-noise, differential-input sensors; PPG: consider motion artifacts; (iv) EMG: specific amplification and filtering; and ensure isolation and safety.
- *Initial review before purchasing a sensor.* Consider the following criteria: (i) Frequencies and ranges covered. (ii) Battery life. (iii) Compatible communication and storage. (iv) Ease of integration and support. (v) Cost and availability.

**Figure 3:**
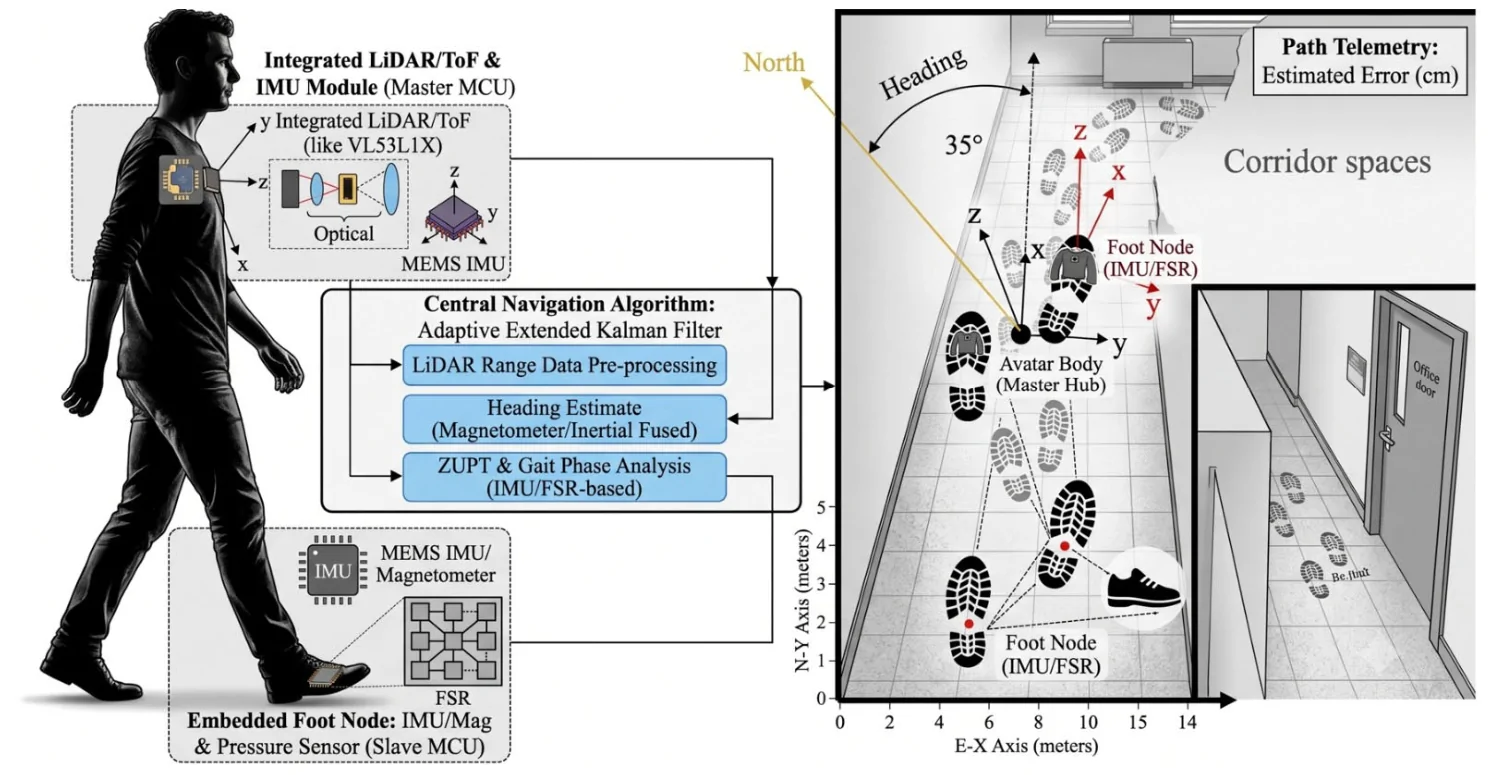
Illustrative wearable body worn sensing architecture for pedestrian positioning and gait-related monitoring, combining a chest-mounted master unit (range/inertial sensing) and a foot node (IMU/magnetometer/FSR) with centralized filtering and path visualization.

Figure 3 illustrates the comprehensive system architecture and corresponding navigation visualization for an integrated pedestrian positioning system utilizing body-worn sensors. The system is founded on a central processing hub that executes an Adaptive Extended Kalman Filter-based Central Navigation Algorithm, receiving inputs from two key sensor nodes. A chest-mounted Integrated LiDAR/ToF & IMU Module (Master MCU) provides global position and orientation data, while an embedded Foot Node (Slave MCU), containing IMU, Magnetometer, and a grid of Force Sensitive Resistors (FSRs), contributes gait phase analysis and ZUPT (Zero-Velocity Update) capabilities. The processed telemetry is visualized on the right in two nested views: a 2D plan view mapping distinct tracks for the Avatar Body (chest unit) and the Foot Node against a meters-scaled grid, showing a 35-degree heading offset from North, and an isometric perspective view showcasing the path within a realistic corridor setting with labeled office spaces, facilitating direct validation of the estimated path against spatial features.

### 4.6. Limitations and Future Perspectives

Although this systematic review provides up-to-date and timely information on the use of microcontrollers and development boards in physical exercise and sports, the results should be interpreted with caution due to the methodological limitations inherent in this type of study. Consequently, the studies included in this document are preliminary in nature, with a predominance of pilot studies, proof-of-concept studies, and instrument validation studies based on small sample sizes. This highlights the scarcity of larger-scale confirmatory studies, which impacts the external validity compared to the precision of the metrological estimates. Furthermore, the complexity of achieving a quantitative synthesis of homogeneous characteristics across the various sports disciplines studied, objectives (development vs. validation), hardware–sensor–firmware combinations, device locations, connectivity, sampling frequencies, and algorithms is acknowledged; this hinders descriptive integration.

Similarly, the included studies rely on controlled approaches in laboratory settings, raising questions about their direct transfer to ecological scenarios (field, competition, variable environmental conditions) in real-world participation settings. Consequently, their applicability remains limited. On the other hand, from a methodological standpoint, the MINORS instrument reveals recurring weaknesses in the included studies: a lack of prospective sample size calculation in many works, inapplicable or unreported loss/follow-up data, and limitations inherent to non-randomized designs. Finally, the difficulty in tracking and applying the appropriate terms in the search formula is acknowledged. It is also noted that the search timeframe is limited, given the rapid evolution of the technological field, which means that subsequent developments are not considered.

Regarding future research, the need for the development of systematic guidelines for validation, calibration, and synchronization of microcontrollers, boards, hardware, firmware, algorithms, and other components is highlighted. Similarly, the formulation of more ecological studies (field/competition) accompanied by longitudinal studies is suggested, including women and a diversity of sporting profiles, and, why not, expanding variables beyond the biomechanical: integrating dimensions of interest for high performance, such as physiological, psychological, and contextual/tactical factors, among others [48,49].

### 4.7. Practical Applications

The main findings allow for the development of practical applications based on low-cost monitoring of strength, power, and velocity-based training (VBT); kinetic and neuromuscular evaluation of countermovement jump (CMJ) and variations with validated DIY platforms; technical analysis and movement correction (IMU + automatic classification); and real-time biofeedback (visual or haptic) for motor learning and technique. Rehabilitation and readaptation with objective measurement (proprioception/balance); monitoring exercise correctness in gym-based strength training (IMU + embedded ML); safety and prevention of physiological risks (fatigue/thermal stress), and technological accessibility for applied research and clubs with limited budget [50].

## 5. Conclusions

In summary, the evidence examined confirms that the use of microcontrollers and development boards for data recording in physical exercise and sports has grown exponentially. It focuses specifically on ESP32 and Arduino, with a strong emphasis on portable and low-cost tools. IMUs are the most used sensors for athlete assessment and control, followed by force sensors/encoders, GPS sensors, and, to a lesser extent, physiological biosensors. Furthermore, regarding the most common biomechanical variables GRF, movement speed, kinematics, and movement classification), several studies report a high level of validity and reliability compared to commercial references or comparable methods, which supports the use of these instruments in the fields of physical exercise and sports.

However, the field is in an early stage of consolidation characterized by small sample sizes, pilot designs, and validations in controlled tasks, with frequent limitations in the reporting of calibration, synchronization, and standardized protocols. Consequently, while these technological advances show significant potential for democratizing sports instrumentation and enabling even real-time feedback or edge processing, their widespread adoption requires strengthening ecological validity and clarifying methodological rigor. This is further supported by promoting reproducibility through comprehensive technical descriptions and, where possible, open datasets.

## Figures and Tables

**Table 1:** Inclusion and exclusion criteria for study selection.

Population	Intervention	Outcomes
People in a sports or physical exercise context: athletes, practitioners of physical activity; subjects evaluated in biomechanics; users of “body-worn” sensors (wearables).	Use of embedded platforms/microcontrollers for instrumentation and monitoring: microcontroller, Arduino, ESP32, Raspberry Pi, STM32, or embedded system, development board applied to sport/physical activity (e.g., integration with IMU/portable sensors).	Data acquisition and transmission/management in athletes and in sport: data acquisition, monitoring, telemetry, real-time, Internet of Things (IoT); movement/biomechanical metrics with inertial measurement unit (IMU)/wearable sensors (activity tracking, kinematic variables, etc.).

Note: IMU, inertial measurement unit; IoT, Internet of Things.

**Table 2:** Methodological quality of the included studies (MINORS).

Reference	I 1	I 2	I 3	I 4	I 5	I 6	I 7	I 8	I 9	I 10	I 11	I 12	Points
Wang et al. [25]	2	1	2	2	2	2	0	0	NA	NA	NA	NA	11
Segura-Garcia et al. [26]	2	1	2	2	0	1	0	0	NA	NA	NA	NA	8
Hsu et al. [27]	2	2	2	2	2	2	0	1	NA	NA	NA	NA	13
Örücü and Selek [28]	2	1	2	2	2	2	0	0	2	1	1	1	16
Mohammed et al. [29]	2	2	2	2	0	2	0	0	NA	NA	NA	NA	10
Hsu et al. [30]	2	2	2	2	2	2	0	0	NA	NA	NA	NA	12
Rusdiana et al. [31]	2	1	2	2	1	2	0	0	1	2	1	2	16
Crandall et al. [32]	2	2	2	2	0	2	1	0	NA	NA	NA	NA	11
Lian et al. [33]	2	1	0	2	2	2	0	0	1	1	0	1	12
Navarro-Iribarne et al. [34]	2	0	0	2	0	2	0	0	NA	NA	NA	NA	6
Duran Bernardes and Ozbay [35]	2	2	0	2	1	2	0	0	NA	NA	NA	NA	9
Smiley et al. [36]	2	0	2	2	2	2	0	0	NA	NA	NA	NA	10
Tan et al. [37]	2	1	2	2	2	2	0	0	NA	NA	NA	NA	11
Wang et al. [38]	2	0	2	2	2	2	0	0	NA	NA	NA	NA	10
Miguel et al. [39]	2	1	1	2	1	1	0	0	NA	NA	NA	NA	8
Pousibet-Garrido et al. [40]	2	1	2	2	1	1	0	0	NA	NA	NA	NA	9
Rashma et al. [41]	2	1	1	2	0	2	0	0	2	1	0	1	12
Kos et al. [42]	2	1	1	2	2	2	2	1	NA	NA	NA	NA	13
Dobra et al. [43]	2	0	2	2	0	2	0	0	NA	NA	NA	NA	8
Dobra et al. [44]	2	0	2	2	0	2	0	0	NA	NA	NA	NA	8
Gunawan et al. [45]	2	1	2	2	1	2	0	0	NA	NA	NA	NA	10
Duarte et al. [46]	2	1	2	2	1	2	0	0	NA	NA	NA	NA	10
Fay et al. [47]	2	0	2	2	2	2	0	0	NA	NA	NA	NA	10

Note. I: Item; Item 1; clearly stated objective, Item 2; inclusion of consecutive patients, Item 3; prospective data collection, Item 4; outcomes appropriate to the study objective, Item 5; unbiased assessment of the study outcome, Item 6; follow-up period appropriate to the study objective, Item 7; loss to follow-up less than 5%, Item 8; prospective calculation of study size, Item 9; adequate control group, Item 10; contemporaneous groups, Item 11; baseline equivalence of groups, Item 12; appropriate statistical analyses. NA; not applicable.

**Table 3:** Main characteristics of the included studies.

Reference	Country	Discipline/Context	Sample (N)	Principal Objective
Wang et al. [25]	China	Swimming	8 swimmers	Analyze stroke technique and swimming phases using accelerometers.
Segura-García et al. [26]	Spain	Soccer	14 players	Build and validate a low-cost GPS/IMU system for external loads.
Hsu et al. [27]	Taiwan	Multi-sport activity classification	NR in the manuscript	Classify sport activities using wearable inertial sensing and a deep convolutional neural network.
Örücü and Selek [28]	Turkey	Athletics	NR (50 pitches)	Design and validate a multichannel wireless wearable surface electromyography (SEMG) system for real-time training performance monitoring.
Mohammed et al. [29]	Iraq	Running/Health	5 subjects	Evaluate vital signs and health alerts.
Hsu et al. [30]	Taiwan	Multi-sport activity classification	NR in the manuscript	Develop a wearable inertial sensing system and a deep CNN to classify sport activities.
Rusdiana et al. [31]	Indonesia	Swimming	46 students/athletes	Biomechanical description of pre-competition starts.
Crandall et al. [32]	USA	Ski	1 subject	Distributed system for ski dynamics (SkiMon).
Lian et al. [33]	China	Basketball	NR in the manuscript	Recognize player identity and basketball shot types from shooting-motion data using an NN-enhanced IoT wristband.
Navarro-Iribarne et al. [34]	Spain	Athletics (discus throw)	NR; amateur discus throwers (case study)	Design and evaluate a low-cost wearable system to measure and graph 3D wrist kinematics during discus throwing.
Duran Bernardes and Ozbay [35]	Portugal	Cycling	8 cyclists	Integrate and evaluate health and power variables in real time.
Smiley et al. [36]	USA	Home telerehabilitation/ cycling	11 healthy adults (9 monitored; 2 controlled)	Design and test low-cost wireless interfaces for remote monitoring and control of cycling exercise during telerehabilitation.
Tan et al. [37]	China	Badminton	9 university students	Design and evaluate a distributed badminton agility training and test system based on an embedded system and Android.
Wang et al. [38]	China/Canada	Hammer throw	1 athlete	Develop a wearable AI-assisted system for real-time biomechanical feedback in hammer throw.
Miguel et al. [39]	Portugal	Cycling and hiking trails/ outdoor physical activity	Proof-of-concept prototype; no formal athlete sample	Design and validate a low-cost IoT system to detect, classify, and count trail users.
Pousibet-Garrido et al. [40]	Spain	Cross-country skiing	NR in the manuscript	Classify skating cross-country skiing gears using inertial sensors and deep learning.
Rashma et al. [41]	Jordan	Rehabilitation	20 subjects	Design, manufacture, and build a technological device for physical therapy.
Kos et al. [42]	Slovenia	Tennis	5 players	Categorize technical actions with low energy consumption.
Dobra et al. [43]	Romania	Swimming	2 adult subjects	Develop a dryland swimming-training system with embedded devices and compare DTW and PCC for technical gesture matching.
Dobra et al. [44]	Romania	Swimming	1 swimmer	Develop and evaluate a wearable DTW-based system for real-time correction of front-crawl strokes in water.
Gunawan et al. [45]	Indonesia	Shorinji Kempo/Martial arts	NR (70 kick samples)	Develop and evaluate an IMU-based dataset and classification system for kick types.
Duarte et al. [46]	Portugal	Strength training/weightlifting	8 participants (6 male, 2 female)	Classify correct vs. incorrect gym exercise execution in real time using an embedded system attached to the weight.
Fay et al. [47]	Australia	Women’s contact sport/ rugby	4 female participants (2 with tackling data)	Develop and preliminarily validate a portable wireless wearable system for monitoring localized breast impacts in female athletes.

Note: NR: not reported; AI, artificial intelligence; IoT, Internet of Things; VBT, velocity-based training; IMU, inertial measurement unit; DTW, dynamic time warping; PCC, Pearson correlation coefficient; GPS, global position system; CNN, convolutional neural network.

**Table 4:** Technical characteristics and main contributions of embedded systems (microcontrollers and sensors) for performance measurement and analysis.

Reference	Principal Microcontroller	Integrated Sensors	Measured Variables	Results/Main Contribution
Wang et al. [25]	TI/Arduino	Triaxial accelerometer	Stroke Rate	Robust algorithm for stroke detection in swimming
Segura-García et al. [26]	Arduino Uno/Mini	GPS, MPU9250	Position, Velocity, G-force	<5% error in total distance vs. commercial systems
Hsu et al. [27]	Arduino Pro Mini (ATmega328)	6-axis IMU (MPU-6050)	Triaxial acceleration, angular velocity	Recognized 10 sports with Acc = 99.86% and CCR = 99.30% (10-fold CV)
Örücü and Selek [28]	Arduino Uno	Laser, ultrasound	Distance (m)	Eliminates subjectivity in throw measurement
Mohammed et al. [29]	Arduino Mega	HR, Temp, BT	Heart Rate, °C	Fatigue and heat stroke prevention system
Hsu et al. [30]	Wearable inertial sensing platform	Inertial sensors	Sport-activity class	Deep-CNN-based classification of sport activities; all hardware details must be verified against the cited full text.
Rusdiana et al. [31]	Arduino Nano	Load cell, BT	Force, T Reaction	Portable system suitable for kinetic analysis in swimming pools
Crandall et al. [32]	Adafruit Feather	IMU, Flex, GPS	Board Flexion, Velocity	Modular architecture for snow research
Lian et al. [33]	IoT wristband	Inertial motion sensors	Player identity and shot type	Neural-network recognition of basketball player identity and shot types from shooting motion.
Navarro-Iribarne et al. [34]	Arduino Nano 33 BLE	9-axis IMU (LSM9DS1)	3D trajectory, speed, orientation	Wearable biofeedback system for real-time wrist tracking in discus throwing
Duran Bernardes and Ozbay [35]	Raspberry Pi/STM32	Pulse oximeter, IMU	HR, SpO_2_, Cadence	Complex embedded system for cyclist health
Smiley et al. [36]	FireBeetle ESP32	Reed switch/hall sensor/ pulse oximeter	Cadence (RPM), HR, SpO_2_	Adequate accuracy for home telerehabilitation; low mean RPM differences vs. tachometer in BLE and Wi-Fi modes.
Tan et al. [37]	STC89C52	Photoelectric sensor	Reaction time, agility	Distributed wireless badminton agility system with significant reaction-time improvements after 8 weeks (*p* < 0.01).
Wang et al. [38]	Arduino Pro Mini	2 IMUs + load cell	Tension, joint angles	0.87% load-cell error and 6% IMU accuracy enabled real-time biomechanical feedback.
Miguel et al. [39]	Raspberry Pi 4 Model B	Camera Module 3	User class, count, timestamp	Embedded computer-vision prototype using YOLOv3-Tiny and smartphone bridge nodes for delayed upload.
Pousibet-Garrido et al. [40]	Embedded inertial-sensing platform	Inertial sensors	Cross-country skiing gear	Deep-learning classification of skating cross-country skiing gears.
Rashma et al. [41]	ESP32	MPU6050 (IMU)	Balance Angles	Effective biofeedback for proprioceptive rehabilitation
Kos et al. [42]	ESP32-S3	IMU (LSM6DS3)	6-Axis Movement	High energy efficiency for extended use on the track
Dobra et al. [43]	ESP32 Lolin Lite	3 × MPU6050 IMUs (accelerometer data used)	Triaxial acceleration/ gesture similarity	Low-cost dryland front-crawl monitoring system; DTW was more robust than PCC for stricter and cross-subject matching.
Dobra et al. [44]	ESP32 Lolin Lite	3 × MPU6050 IMUs (accelerometer + gyroscope)	Triaxial acceleration/ angular velocity/ stroke similarity	Low-cost wearable system for real-time stroke correction in water using DTW and vibration feedback.
Gunawan et al. [45]	ESP32	MPU6050 IMUs	3-axis angular velocity and linear acceleration	SVM reached 96.7% accuracy with 70 samples and two sensors; k-NN reached 92.4%; RF reached 90.0%.
Duarte et al. [46]	Seeed Studio XIAO nRF52840 Sense	LSM6DS3 IMU (accelerometer + gyroscope)	Acceleration, angular velocity, exercise correctness	Off-the-person HAR system for weights with real-time classification of correct vs. incorrect execution; best reported accuracy 94.1%.
Fay et al. [47]	Raspberry Pi Pico W (RP2040)	16 thin-film FSRs	Localized breast impact force, spatial distribution	Portable wireless breast-impact monitoring system validated with calibration R^2^ = 0.9988 and preliminary impacts up to 550 N.

Note: GPS, global positioning system; IMU, inertial measurement unit; TI, Texas Instruments; BT, Bluetooth; HR, heart rate; Temp, temperature; FSR, force-sensitive resistor; SpO_2_, peripheral oxygen saturation; MPV, mean propulsive velocity; DTW, dynamic time warping; PCC, Pearson correlation coefficient; SVM, support vector machine; k-NN, k-nearest neighbors; RF, random forest; MPU, motion processing unit; RPM, revolutions per minute; R^2^, coefficient of determination; m, meters.

**Table 5:** Overview of Features and Models.

Reference	Sensors	Variables	Contribution for the Professional
Wang et al. [25]	Accelerometer	Stroke frequency	Detailed analysis of swimming efficiency per stroke cycle
Segura-García et al. [26]	GPS/MPU9250	Distance/Speed	External load monitoring with <5% error compared to the gold standard
Hsu et al. [27]	6-axis IMU (accelerometer + gyroscope)	Acceleration/angular velocity	Automatic classification of 10 sport activities using wearable sensors on the dominant wrist and ankle
Örücü and Selek [28]	Laser/Ultrasonic	Linear distance	Digitization and improved precision in athletics field testing
Mohammed et al. [29]	HR/Temp/BT	BPM/Body temperature (°C)	Real-time prevention of physiological risks
Hsu et al. [30]	Wearable inertial sensors	Acceleration/angular velocity/sport class	Deep-CNN classification of multiple sport activities.
Rusdiana et al. [31]	Load cell/BT	Force/Time	Low-cost kinetic evaluation system for swimming
Crandall et al. [32]	Multi-IMU/GPS	Flexion/Dynamics	Quantification of sports equipment material deformation
Lian et al. [33]	Wristband inertial sensors	Basketball shooting motion	Recognition of player identity and shot types using a neural-network-enhanced IoT wristband.
Navarro-Iribarne et al. [34]	9-axis IMU	3D kinematics/hand speed	Immediate technical feedback on throw path and hand speed
Duran Bernardes and Ozbay [35]	Pulse oximeter/IMU	SpO_2_/Cadence	Complex embedded system for monitoring health and fatigue in cycling
Smiley et al. [36]	Reed switch/hall sensor/ pulse oximeter	RPM/HR/SpO_2_	Remote supervision and control of cycling exercise in home rehabilitation with simple visual feedback and acceptable metrological accuracy
Tan et al. [37]	Photoelectric sensors	Reaction time/agility	Objective multi-point badminton agility training and on-court response-time evaluation
Wang et al. [38]	Load cell/2 IMUs	Tension/joint angles	Real-time synchronization of kinetic and kinematic feedback for hammer-throw training
Miguel et al. [39]	Camera/smartphone bridge node	User type, counts, peak use	Objective route-use monitoring for infrastructure management, tourism, and outdoor activity planning.
Pousibet-Garrido et al. [40]	Inertial sensors	Skiing gear/movement class	Deep-learning classification of skating cross-country skiing gears.
Rashma et al. [41]	IMU (MPU6050)	Balance/Angles	Objective biofeedback system for proprioceptive rehabilitation
Kos et al. [42]	IMU (LSM6DS3)	6-axis movement	Low-power device for long-term monitoring
Dobra et al. [43]	3 × MPU6050 IMUs	Acceleration/ pattern similarity	Dryland front-crawl monitoring with immediate technique assessment and better transfer across subjects when DTW is used.
Dobra et al. [44]	3 × MPU6050 IMUs	Acceleration/ angular velocity/ DTW similarity	Immediate vibrotactile feedback for front-crawl technique correction and remote monitoring in water.
Gunawan et al. [45]	MPU6050 IMUs	Angular velocity/linear acceleration/kick pattern	Automatic classification of martial arts kicks with wearable sensors in field-like conditions
Duarte et al. [46]	6-axis IMU	Execution correctness/ movement classification	Real-time feedback on weightlifting techniques with a compact low-cost device attached to the weight.
Fay et al. [47]	16 thin-film FSRs	Localized breast impact force/distribution	Objective monitoring of local breast loads and evaluation of protective equipment in female contact sport.

Note: IMU; Inertial Measurement Unit, GPS; Global Positioning System, GRF; Ground reaction force, VBT; Velocity-based training, HR; heart rate, Temp; Temperature, BT; Bluetooth; BPM; Beats per minute, SpO_2_; Peripheral oxygen saturation, DTW; Dynamic Time Warping, AI; Artificial intelligence.

**Table 6:** Biomechanical/functional variables measured and main results.

Reference	Application/Discipline	Main Variable Evaluated	Performance Metrics/Key Results
Wang et al. [25]	Swimming	Stroke rate and style	Detection sensitivity: 96.8%.
Segura-García et al. [26]	Soccer	Distance and speed (GPS)	Relative error < 5.2% vs. commercial device.
Hsu et al. [27]	10 sport activities	Triaxial acceleration and angular velocity	CCR: 99.30% (10-fold); 98.20% (LOSO).
Örücü and Selek [28]	Athletics	Throw distance (Shot)	Millimeter precision; reduced human error.
Mohammed et al. [29]	Running/Health	Heart rate and body temperature	Wireless signal stability during riding.
Hsu et al. [30]	Multi-sport activity classification	Sport-activity recognition	Deep-CNN-based classification; numerical performance metrics must be checked against the cited full text.
Rusdiana et al. [31]	Swimming (Exit)	Reactive and kinematic strength	Reaction time and peak kinetic force.
Crandall et al. [32]	Ski	Ski flexion and deformation	Successful multi-node synchronization.
Lian et al. [33]	Basketball	Player identity and shot-type recognition	Neural-network recognition based on basketball shooting-motion analysis; numerical metrics must be verified.
Navarro-Iribarne et al. [34]	Athletics (discus throw)	3D wrist trajectory/speed	Functional validation: real-time plots reconstructed throw phases and direction changes.
Duran Bernardes and Ozbay [35]	Cycling	SpO_2_ and cadence	Successful integration into a DIY cycling computer.
Smiley et al. [36]	Home telerehabilitation/ cycling	Cadence accuracy (RPM) + HR/SpO_2_	Mean RPM difference vs. tachometer: 0.38 ± 0.03 (BLE), 0.25 ± 0.27 (Wi-Fi), and 6.7 ± 3.3 (motorized Bluetooth).
Tan et al. [37]	Badminton	Combined reaction time across eight court directions	Significant reductions in reaction time after 8 weeks (*p* < 0.01).
Wang et al. [38]	Hammer throw	Wire tension, vertical displacements, joint angles	Load-cell error: 0.87%; IMU accuracy: 6%; complete model MAE ~4–5° for lower-limb angles.
Miguel et al. [39]	Cycling and hiking trails	User detection and count	YOLOv3-Tiny mAP: 78.4%; 12 detections were transferred successfully to the central database in field validation.
Pousibet-Garrido et al. [40]	Cross-country skiing	Skating gear classification	Deep-learning gear classification using inertial sensors; numerical metrics must be verified.
Rashma et al. [41]	Rehabilitation	Balance angles (Roll/Pitch)	Significant improvement in postural control (*p* < 0.05).
Kos et al. [42]	Tennis	Gesture recognition	F1-Score: >0.90; Low power consumption.
Dobra et al. [43]	Swimming	Front-crawl stroke similarity (dryland accelerometry)	Good-stroke examples reached 91% (DTW) and 93% (PCC); incorrect movements fell to 58% and 42%. An 80% threshold was used, and DTW performed better for subject transfer.
Dobra et al. [44]	Swimming	Front-crawl stroke similarity (acceleration + gyroscope)	In-water similarity: 27%, 23%, and 86%; dryland-to-water: 59%, 73%, and 83%. Vibration activated when similarity < 70%.
Gunawan et al. [45]	Shorinji Kempo/Martial arts	Kick-type classification	SVM: 96.7% (2 sensors); k-NN: 92.4%; RF: 90.0% with 70 processed samples.
Duarte et al. [46]	Strength training/weightlifting	Correct vs. incorrect execution of gym exercises	Edge Impulse accuracy ranged from 72.2% to 94.1%; TensorFlow Lite results were around 59–65%.
Fay et al. [47]	Women’s contact sport/ rugby	Localized breast impact force and distribution	856 Hz per channel; calibration R^2^ = 0.9988; preliminary impacts up to 550 N during simulated tackling.

Note: CCR: correct classification rate; LOSO: leave-one-subject-out; SVM: support vector machine; RPM: revolutions per minute; HR: heart rate; SpO_2_: peripheral oxygen saturation; mAP: mean average precision; MPV: mean propulsive velocity; MAE: mean absolute error; DTW: Dynamic Time Warping; PCC: Pearson correlation coefficient; BLE: Bluetooth Low Energy; DIY: do-it-yourself; k-NN: k-nearest neighbors; RF: random forest.

**Table 7:** Experimental implementation and operational feasibility.

Reference	Microcontroller (MCU)	Sensors Used	Device Location	Connectivity/Frequency	Software/Algorithm
Wang et al. [25]	TI MCU (Arduino IDE)	Triaxial accelerometer	Wrist	Bluetooth/50 Hz	Zero-crossing algorithm
Segura-García et al. [26]	Arduino Uno/Mini	GPS (Ublox) + IMU	Upper back	SD card/10 Hz	Post-processing analysis
Hsu et al. [27]	Arduino Pro Mini (ATmega328)	MPU-6050 (accelerometer + gyroscope)	Dominant wrist and ankle	RF (nRF24L01)/100 Hz	STFT spectrogram + CNN
Örücü and Selek [28]	Arduino Uno	Laser + Ultrasonic	Fixed station	Wired (Serial)	Linear calculation algorithm
Mohammed et al. [29]	Arduino Mega	MAX30100 + LM35	Wrist/Chest	Bluetooth/N/A	Threshold-based alerts
Hsu et al. [30]	Wearable inertial platform	Inertial sensors	Wrist/body-worn	Wireless; verify frequency	Deep convolutional neural network
Rusdiana et al. [31]	Arduino Nano	TAL220 Load Cell	Starting platform	Bluetooth/100 Hz	Android app (processing)
Crandall et al. [32]	Adafruit Feather	IMU + Flex Sensors	Ski surface	BLE/100 Hz	NTP synchronization
Lian et al. [33]	IoT wristband	Inertial motion sensors	Wrist	Wireless; verify frequency	Neural network for identity and shot-type recognition
Navarro-Iribarne et al. [34]	Arduino Nano 33 BLE	IMU (LSM9DS1)	Wrist	BLE/476 Hz	Madgwick fusion + quaternion processing
Duran Bernardes and Ozbay [35]	Raspberry Pi/STM32	SpO_2_ + IMU	Handlebar	ANT+/BLE	Real-time data integration
Smiley et al. [36]	FireBeetle ESP32	Reed switch/hall sensor/ WristOx2 pulse oximeter	Portable bike and motorized bike (frame/pedal area)	BLE or Wi-Fi; classic Bluetooth/real time	C# tablet app + C++ interval detection and remote control
Tan et al. [37]	STC89C52 + ESP8266	PNP-NC photoelectric sensor	Center and eight court stations	Wi-Fi/NR	Android app + Java Socket
Wang et al. [38]	Arduino Pro Mini 328-5V	2 × IMUs + load cell	Waist-mounted node/wrist/hammer handle	XBee 802.15.4/50 Hz	Madgwick filter + deep neural networks
Miguel et al. [39]	Raspberry Pi 4 Model B	Camera Module 3	Fixed node beside trail	Bluetooth 5.0 to Android bridge node; Internet sync via smartphone/NR	YOLOv3-Tiny CNN + opportunistic data transfer
Pousibet-Garrido et al. [40]	Embedded sensing platform	Inertial sensors	Skier/body-worn	Verify in full text	Deep learning for gear classification
Rashma et al. [41]	ESP32	IMU (MPU6050)	Center of the board	Wi-Fi/50 Hz	Kalman filter/Mobile app
Kos et al. [42]	ESP32-S3	IMU (LSM6DS3)	Dominant wrist	BLE/104 Hz	Random Forest/Edge AI
Dobra et al. [43]	ESP32 Lolin Lite (1 master + 3 slaves)	3 × MPU6050 IMUs	Wrist, elbow, and shoulder of one arm	ESP-NOW + Wi-Fi/ 50 ms sampling (~20 Hz)	Dynamic Time Warping (DTW) vs. PCC
Dobra et al. [44]	ESP32 Lolin Lite	3 × MPU6050 IMUs	Left-arm wrist, elbow, and shoulder	ESP-NOW + Wi-Fi/ 50 ms sampling (~20 Hz)	Dynamic Time Warping (DTW) + vibration feedback
Gunawan et al. [45]	ESP32	2–3 × MPU6050 IMUs	Thigh, shanks, and foot	Wi-Fi/UDP/1 kHz	SVM/k-NN/RF
Duarte et al. [46]	Seeed Studio XIAO nRF52840 Sense	LSM6DS3 IMU (6-axis)	Attached to the weight/dumbbell	BLE/50 Hz	1D CNN (Edge Impulse/TensorFlow Lite)
Fay et al. [47]	Raspberry Pi Pico W (RP2040)	16 thin-film FSRs	On both breasts + GPS-bra rear pocket	BLE/856 Hz per channel	Dual-core MicroPython + calibration model/RBF heat maps

Note: MCU: microcontroller unit; TI: Texas Instruments; IDE: Integrated Development Environment; IMU: Inertial Measurement Unit; RF: radio frequency when referring to connectivity, or random forest when referring to the algorithm; STFT: short-time Fourier transform; CNN: convolutional neural network; BLE: Bluetooth Low Energy; NTP: Network Time Protocol; GPS: Global Positioning System; SD: Secure Digital; ANT+: low-power wireless communication protocol commonly used in sport sensors; DTW: Dynamic Time Warping; PCC: Pearson correlation coefficient; ESP-NOW: low-latency wireless protocol for ESP devices; UDP: User Datagram Protocol; k-NN: k-nearest neighbors; FSR: force-sensitive resistor; RBF: radial basis function; NR: not reported; 1D CNN: one-dimensional convolutional neural network.

## Data Availability

The data presented in this document can be verified based on the results obtained, which can be consulted through the corresponding authors.

## Supplementary Materials

The following supporting information can be downloaded at: https://www.mdpi.com/article/10.3390/s26165027/s1, PRISMA 2020 Checklist.
